# Awareness About Keratoconus and Its Relation With Eye Rubbing: A Cross‐Sectional Study in Riyadh

**DOI:** 10.1002/hsr2.72006

**Published:** 2026-03-29

**Authors:** Omar Alhadlaq S, Albatool Falah Aljofan, Haya Alshutayli, Sara Alnowaiser, Amirah M. Almokhtar, Shaker O. Alreshidi, Taghreed Alnahedh

**Affiliations:** ^1^ College of Medicine King Saud Bin Abdulaziz University for Health Sciences Riyadh Saudi Arabia; ^2^ College of Medicine Majmaah University Majmaah Saudi Arabia; ^3^ College of Medicine Royal College of Surgeons Dublin Ireland; ^4^ Ophthalmology Resident Ministry of National Guard Health Affairs Riyadh Saudi Arabia; ^5^ Ophthalmology Division, Department of Surgical Specialities, College of Medicine Majmaah University Majmaah Saudi Arabia; ^6^ Department of Medical Education, College of Medicine King Saud Bin Abdulaziz University for Health Sciences (KSAU‐HS) Riyadh Saudi Arabia

**Keywords:** allergy, awareness, eye rubbing, keratoconus, Saudi Arabia

## Abstract

**Background:**

Keratoconus is a progressive eye disease affecting the cornea, the clear dome‐ shaped structure at the front of the eye. In keratoconus, the cornea thins and bulges outward into a cone shape, leading to loss of visual acuity. The aetiology of keratoconus remains unclear, but it involves both genetic and environmental factors. Eye rubbing is a well‐established risk factor for keratoconus. Previous studies in Saudi Arabia highlighted a concerning lack of awareness about keratoconus among the general population.

**Aim:**

This study aimed to assess the awareness of keratoconus and its relationship with eye rubbing among the population in Riyadh, Saudi Arabia.

**Methods:**

A cross‐sectional study was performed in Riyadh, Saudi Arabia, involving 341 participants using an online pre‐designed questionnaire from August to September 2024.

**Results:**

A total of 341 participants completed the questionnaire. Participant age ranged from 18 to > 60 years. More than half of the participants (*n* = 215; 63%) were female. Regarding the overall awareness about keratoconus, 102 participants (29.9%) demonstrated a good awareness level; the most common source of information reported was social media (20%). Notably, 276 participants (81%) reported rubbing their eyes because of allergies (8%), headache or fatigue (28%), and itching (58%).

**Conclusion:**

The present study in Riyadh indicated that many participants had a poor awareness of keratoconus and its potential relationship with eye rubbing. It is essential to enhance public awareness through health education programmes.

## 1 Introduction

Keratoconus is a progressive condition affecting the cornea that presents bilaterally and eventually leads to visual deterioration and blindness. It is characterised by ectasia, thinning, and increased curvature of the cornea, resulting in a cone‐like bulge, which causes irregular astigmatism and poor vision, especially with higher‐order aberrations [1, 2].

The estimated prevalence of keratoconus varies significantly among different countries. In 1986 [3], reported an incidence of 2 cases per 100,000 in Minnesota, USA, whereas, in 2017, Goddefrooij et al. reported an incidence of 265 per 100,000 in the Netherlands. In the Middle East, it affects up to 5% of the population [4]. Studies conducted in Asia and the Middle East suggest that keratoconus is prevalent at a younger age in these regions compared to North American and European studies [5]. Assiri et al. reported an incidence rate of 20 cases per 100,000 annually, with many instances exhibiting severe presentations and early onset [5]. Similarly, another study in Medina by Kordi et al. documented a prevalence of 13.2% among ophthalmic patients screened for refractive errors [6]. Another study in Iran highlighted that keratoconus often manifests at a younger age, correlating with environmental factors such as high UV exposure and genetic predisposition [7]. Similarly, research in India revealed that approximately 3.5% of ophthalmic patients screened for refractive errors were diagnosed with keratoconus, further supporting the high prevalence in Asian populations [8].

This discrepancy may be attributed to the fact that the prevalence of keratoconus tends to increase in areas with dry and warm weather owing to high exposure to sunlight, which acts as a contributing factor to its pathogenesis [7]. However, the exact aetiology of the disease remains unclear; it is likely caused by a combination of several factors, including biochemical and genetic factors and mechanical triggers [9].

Studies have shown a higher concordance of keratoconus in identical twins than non‐identical twins. Familial aggregation studies add to this genetic predisposition since first‐degree relatives of keratoconus patients are more likely to develop the disease than the normal population [10]. Genome‐wide association studies have identified specific loci, such as those near the VSX1 and COL4A4 genes, that are associated with increased susceptibility to keratoconus [9]. These results show that genetic factors are involved in the development of keratoconus and that environmental factors, including eye rubbing and UV exposure, may worsen the disease. Moreover, ethnicity is a contributing factor, with Asians experiencing an earlier onset and a more severe form of the disease than Scandinavian [11]. Keratoconus is also triggered by environmental factors, especially in genetically predisposed populations; eye rubbing, systemic allergies, and exposure to UV light have been considered contributing factors [11]. Eye rubbing, a repetitive behaviour triggered by ocular irritation, exhaustion, or emotional stress [12], is considered a significant external factor that induces mechanical changes in the cornea, often acting as the second hit in the two‐hit hypothesis for keratoconus [13]. According to the two‐hit hypothesis, the first ‘hit’ is a genetic predisposition or inherent weakness in the corneal structure, and the second ‘hit’ is an external environmental or mechanical factor, such as chronic eye rubbing, which exacerbates corneal instability and contributes to the progression of keratoconus [13]. Chronic and excessive eye rubbing often follows bothersome symptoms such as dryness and irritation [11]. Allergies and atopy are the most common reasons for the development of this chronic habit [11]. Studies suggest a link between keratoconus and atopy; itchiness increases the incidence of eye rubbing, which causes mechanical wear and tear of the cornea, leading to progressive corneal bulging [8].

Keratoconus manifests with a range of visual complaints, including progressive blurring of vision and potential for significant decline in visual acuity. Notably, asymmetric presentations are observed in approximately 41% of patients at diagnosis, with one eye exhibiting more advanced and severe symptoms. Keratoconus is further characterised by distinct ophthalmic signs, such as anterior and posterior corneal elevation; corneal thinning; and irregular, often skewed, corneal astigmatism with high central keratometry readings [14, 15, 16]. The progression of keratoconus, if not prevented, usually leads to impaired visual acuity. In advanced cases of keratoconus, the only option is corneal transplant. Therefore, early diagnosis is crucial [9, 17].

The key finding of a large‐scale, 8‐year study that analysed over 1200 patients was that 50% of the participants admitted to habitually rubbing their eyes vigorously, which strongly supports the association between eye rubbing and the development of keratoconus [18]. A study conducted in the eastern province of Saudi Arabia that investigated the level of awareness of keratoconus among the general population (*N* = 388) reported that only 26% had an adequate level of awareness of keratoconus, and 81.4% engaged in eye rubbing [19]. A similar study conducted in Aseer province by Al‐Amri et al. [20] reported that 59% of respondents were aware of keratoconus as a disease but only 22.2% had a good level of understanding of the disease.

## 2 Materials and Methods

### 2.1 Study Design and Setting

The present study was a cross‐sectional study of participants older than 18 years in Riyadh, Saudi Arabia. Data were collected through an online questionnaire in Arabic, which was distributed through social media over a 3‐week period in August 2024.

### 2.2 Questionnaire Development and Distribution

A pre‐designed questionnaire in Arabic from a previous study conducted in Medina, Saudi Arabia, was used with permission from the corresponding author. The Arabic version has been previously validated by Kordi et al. (2020). The questionnaire was structured and divided into three sections. The first section included questions about sociodemographic characteristics such as age, sex, education, and marital status. The second section comprised yes/no questions regarding allergic and ocular histories. The last section included 11 items to evaluate the participants' level of awareness of keratoconus, treatment, and causes of eye rubbing.

A minimum required sample size of 341 participants was estimated using Raosoft software [21] (Inc., Seattle, WA, USA), with a 95% confidence level and 5% margin of error.

Personal contacts and various social media platforms were used to ensure broad participation, allowing engagement of different demographics across Riyadh.

### 2.3 Informed Consent and Ethical Approval

Participants were informed about the study objectives, and informed consent was obtained before participation. Ethical approval was obtained from King Abdullah International Medical Research‐King Saud Bin Abdulaziz University for Health Sciences (KAIMRC KSAU‐HU).

### 2.4 Data Analysis

Following data extraction, the information was reviewed, coded, and entered into IBM SPSS Statistics for Windows (Version 27.0) [22] (Armonk, NY, USA). Two‐tailed tests were applied for all statistical analyses, with a significance threshold of *p* < 0.05. In the knowledge assessment, each correct answer received 1 point, and the overall score was calculated by adding the points obtained for all questions. A score below 60% of the total was classified as poor awareness, whereas a score of 60% or higher was deemed good awareness. Participants who had never heard about keratoconus were considered to have a poor level of awareness.

Descriptive analysis was conducted on all variables, including participants' demographic details, family history of keratoconus, medical history of eye diseases, and sources of knowledge about keratoconus, using frequency and percentage distribution. Cross‐tabulation was employed to assess the distribution of participants' awareness levels on the basis of their personal data and practices. Relationships between variables were examined using the Pearson chi‐square test and the exact probability test for small frequency distributions.

## 3 Results

### 3.1 Sociodemographic Characteristics of Participants

A total of 341 participants completed the study questionnaire; their sociodemographic characteristics are outlined in Table 1. Participant age ranged from 18 years to over 60 years. A majority of participants were female (63%). Regarding marital status, 63.4% identified as single, whereas 36.6% were married. Regarding educational level, 12.9% held a master's or PhD degree, 67.5% had a bachelor's degree, and 19.6% had completed a high school diploma or lower.

**TABLE 1:** Sociodemographic characteristics of study participants.

Personal characteristics	Number	Percentage
**Age in years**		
18–30	201	59%
31–40	39	11.4%
41–50	39	11.4%
51–60	42	12.2%
> 60	20	6%
**Gender**		
Female	215	63%
Male	126	37%
**Educational level**		
Master's/PhD	44	12.9%
Bachelor's degree	230	67.5%
High school or lower	67	19.6%
**Marital status**		
Single	216	63.4%
Married	125	36.6%

When participants were asked about their medical history and their family medical history, 34% reported having allergies, including chest (25.8%), skin (24.8%), eye (20.6%), and digestive allergies (10.3%). Additionally, 180 participants (52.8%) reported having ocular or visual problems, of which 4 reported having keratoconus. Moreover, 21 participants reported having a family member diagnosed with keratoconus. Table 2 showed the participants' responses in further detail.

**TABLE 2:** Medical and family histories of study participants.

Family and medical history	Number	Percentage
**Have an allergy?**		
No	225	66%
Yes	116	34%
**Type of allergy**		
Chest allergy	30	9%
Skin allergy	29	8.5%
Eye allergy	24	7%
Digestive allergy	12	3.5%
Other	21	6%
**Have eye problems?**		
Yes	180	52.8%
No	161	47.2%
**Nature of the problem**		
Refractive error	135	39.5%
Use of contact lenses for vision correction	16	4.6%
Eye surgery	11	3.2%
Keratoconus	4	1.2%
Other	14	4.3%
**Family history of keratoconus**		
No	320	93.8%
Yes	21	6.2%

Regarding awareness, 51% of the participants had heard of keratoconus, and the main source of information for 40% of these was social media. Only 16.3% correctly identified keratoconus. Moreover, 29% believed that keratoconus is associated with allergies, and 41% responded that it can lead to poor eyesight. Most participants (58%) were unaware of the treatment options for keratoconus. Only 39.3% acknowledged that eye rubbing may contribute to the development of keratoconus.

Regarding practices among study participants, 276 participants reported that they rubbed their eyes, and 47% of the participants identified itching as the cause of eye rubbing.

Furthermore, 188 participants reported being exposed to sunlight throughout the day, and 34% of these individuals wore sunglasses.

**FIGURE 1:**
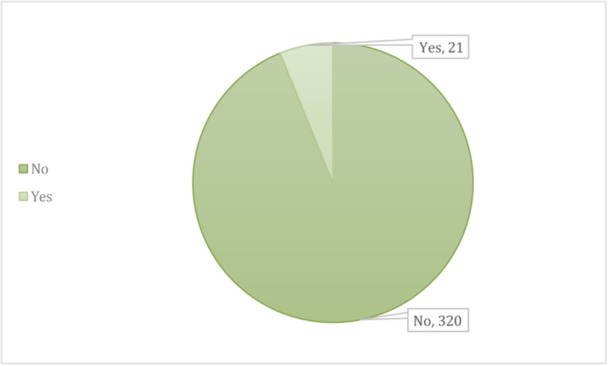
Family history of keratoconus among study participants.

Figure 1 illustrates the family history of keratoconus among participants; only 21 participants reported having a family history, whereas 320 reported no such history.

**FIGURE 2:**
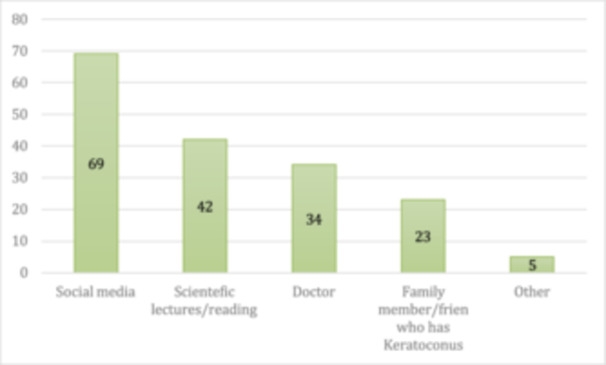
Sources of participants' information about keratoconus.

Figure 2 illustrates the sources of information participants used to learn about keratoconus, with social media being the most common source, followed by scientific lectures and reading materials, healthcare professionals, and finally, family members who have keratoconus. Five participants reported other sources of information.

**FIGURE 3:**
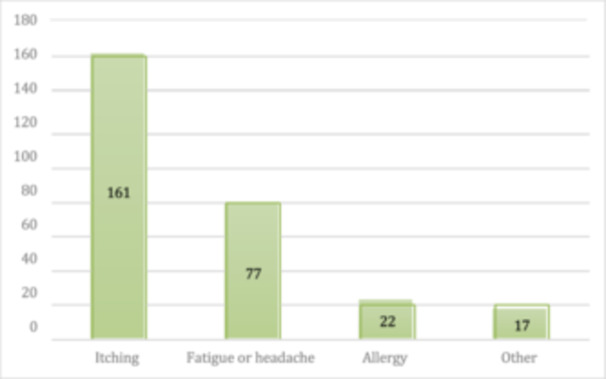
Participants' responses about reasons for eye rubbing.

Figure 3 presents the causes of eye rubbing among participants who reported this behaviour. The most frequent cause was itching, followed by fatigue or headache, allergies, and other reasons (Tables 3 and 4).

**TABLE 3:** Awareness of keratoconus among study participants.

Awareness items	Number	Percentage
**Heard about keratoconus**		
Yes	168	49%
No	173	51%
**Source of information**		
Social media	69	20%
Scientific lectures/reading	42	12.3%
Doctors	34	10%
Relative/friend with keratoconus	23	6.7%
Other	5	2%
**What is keratoconus?**		
I don't know	173	51%
Increased corneal thickness	67	20%
Corneal thinning and weakness	56	16.3%
Corneal inflammation	39	11.4%
Immune disease	6	1.3%
**Is there a relationship between keratoconus and allergy?**		
I don't know	186	55%
Yes	100	29%
No	55	16%
**Does keratoconus lead to poor eyesight?**		
I don't know	181	53%
Yes	141	41%
No	19	6%
**Treatment method for keratoconus**		
I don't know	199	58%
Surgery	73	21.3%
Eyeglasses	21	6.3%
Contact lenses	18	5.2%
Eye drops	16	5%
No treatment	14	4.2%
**The habit of eye rubbing is described as:**		
I don't know	147	43.3%
It may lead to keratoconus	134	39.3%
One of the safe habits	39	11.4%
Other	21	6%

**TABLE 4:** Practices among study participants.

Practices	Number	Percentage
**Do you rub your eyes?**		
Yes	276	81%
No	65	19%
**If yes, why?**		
Allergy	22	6.5%
Fatigue or headache	77	22.5%
Itching	161	47%
Other	16	5%
**Do you get a lot of sun exposure throughout the day?**		
Yes	188	55%
No	153	45%
**If yes, do you wear sunglasses**		
Yes	115	34%
No	73	21%

A comparison of the level of awareness among different groups of study participants showed that there was more awareness among female participants than male participants; however, the difference was not statistically significant. Notably, awareness was significantly higher in participants with a family history of keratoconus (*p* = 0.007; Table 5).

**TABLE 5:** Overall awareness level among study participants.

Awareness level					
Factor			Good	Poor	*p*‐value
Age group	18–30	*n*	37	164	0.383
		%	18.4%	81.6%	
	31–40	*n*	12		
		%	30.8%	69.2%	
	41–50	*n*	9	30	
		%	23.1%	76.9%	
	51–60	*n*	6	36	
		%	14.3%	85.7%	
	Older than 60	*n*	4	16	
		%	20.0%	80%	
Gender	Female	*n*	45	170	0.578
		%	20.9%	79.1%	
	Male	*n*	23	103	
		%	18.3%	81.7%	
Do you have a family history of keratoconus?	No	*n*	57	263	< 0.001*
		%	17.8%	82.2%	
	Yes	*n*	11	10	
		%	52.4%	47.6%	

*Note:* *Indicates statistical significance (*p* < 0.05).

Notably, a significant relationship was observed between having an allergy and having a family history of keratoconus (Table 6).

**TABLE 6:** Association between having an allergy and a family history of keratoconus.

Factor	Do you have family history of keratoconus?			*p*‐value
Do you have an allergy?		Yes, *n* [%]	No, *n* [%]	0.021*
	Yes; *n* [%]	12 [10.3]	104 [89.7]	
	No; *n* [%]	9 [4]	216 [96]	

*Note:* *Indicates statistical significance (*p* < 0.05).

In contrast, there was no significant association between eye rubbing and having a family history of keratoconus (Table 7).

**TABLE 7:** Association between eye rubbing and having a family history of keratoconus.

Factor	Do you have family history of keratoconus?			*p*‐value
Do you rub your eyes?		Yes; *n* [%]	No; *n* [%]	1.0
	Yes, *n* [%]	17 [6.2]	259 [93.8]	
	No, *n* [%]	4 [6.2]	61 [93.8]	

**FIGURE 4:**
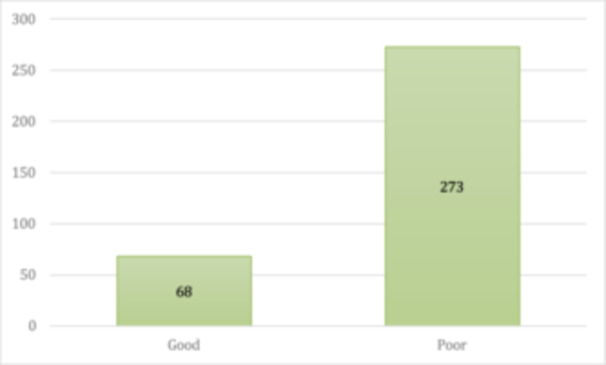
Figure showing awareness levels about keratoconus.

Regarding the overall awareness about keratoconus, 68 participants demonstrated a good awareness level, accounting for approximately 20% of the total respondents. In contrast, 273 (80%) participants exhibited a poor awareness level (Figure 4).

**FIGURE 5:**
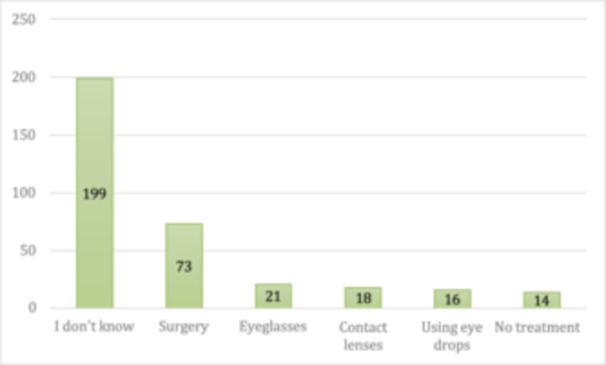
Participants' responses about treatment options for keratoconus.

## 4 Discussion

The present study aimed to investigate the awareness about keratoconus and its association with eye rubbing among the population in Riyadh, Saudi Arabia. The sample size was small; nevertheless, only 1.2% of the participants reported having keratoconus, indicating a lower prevalence of keratoconus than that reported in other regional studies conducted in the eastern province of Saudi Arabia (4.2%) [19] and in Madina (13.2%) [6]. This discrepancy might be explained by the geographical and demographic variations in the effects of keratoconus [23].

The study found higher awareness levels among participants with a family history of keratoconus. A similar level of awareness was reported in the Aseer region by Al‐Amri et al. [20], documenting that 18.7% of respondents had adequate knowledge of keratoconus (Figure 5). Another study in Abha, Saudi Arabia, found that most participants (85.74%) had no prior knowledge of keratoconus, with only 14.26% being aware of the condition through lectures and reading [24]. Similarly, Al Rashed et al. concluded that the overall level of awareness about eye diseases among the public in Saudi Arabia was poor [25]. In terms of treatment, most participants in the present study were aware of surgery as the primary treatment of choice, followed by eyeglasses, contact lenses, and eyedrops. However, over half (58%) were unaware of the treatment methods as they had no prior knowledge about the condition.

Previous studies indicate that the level of knowledge about keratoconus among the general population varies. Nevertheless, our study population showed a decent level of awareness regarding keratoconus. Over half of the study participants, 173 (51%) had previously heard about keratoconus, but only 68 (20%) of these individuals had a good level of awareness. Moreover, their sources of information differed slightly. Social media had the largest impact, with 20% of the participants reporting it as the main source. Other sources of information included scientific lectures, doctors, and family members or friends with a history of keratoconus.

Regarding the association between eye rubbing and keratoconus, 134 (39.3%) of participants believed that eye rubbing may lead to keratoconus. The study by AlSomali et al. reported that a higher percentage of participants (42.3%) believed that eye rubbing could potentially lead to keratoconus. In contrast, other studies have reported a considerably lower level of awareness of the relationship between eye rubbing and keratoconus. Al‐Amri et al. [20] reported that 33.1% of the participants believed that eye rubbing was the most prevalent risk factor associated with keratoconus, whereas Kordi et al. reported that only 28.9% of the participants considered that eye rubbing potentially causes keratoconus.

In the present study, most participants (81%) reported that they rubbed their eyes, but only 47% of the participants reported that this behaviour was related to itching. This finding differed from previous reports; for example, Kordi et al. reported that the majority of participants rubbed their eyes primarily because of itching (71.6%), and AlSomali et al. reported that more than half of the participants (81.4%) rubbed their eyes, with the main cause being itching (66.5%).

A limitation of this study is using an online distribution method as this would limit the reach to elderly and lower socioeconomic status of the population. This Study sample may not be entirely inclusive or representative of the entire population, especially older age groups or individuals of lower socioeconomic status, which may have affected the overall quality of results.

## 5 Conclusion

The study revealed that a large proportion of participants in Riyadh, Saudi Arabia, had poor 326 awareness of keratoconus, with only 29.9% demonstrating a good level of knowledge about 327 the condition. Notably, 81% of participants reported rubbing their eyes, with itching being the 328 most common cause, yet only 39.3% recognised the potential link between eye rubbing and 329 keratoconus. Social media emerged as the primary source of information, highlighting gaps in 330 public education. Considering the high prevalence of keratoconus in Saudi Arabia and its 331 potential association with eye rubbing, targeted health education programmes are crucial to 332 improve awareness and promote preventive measures.

## Author Contributions

Supervision: Omar Alhadlaq. Methodology: Haya Alshutayli, Sara Alnowaiser, Albatool Falah Aljofan. Data curation: Haya Alshutayli, Sara Alnowaiser, Albatool Falah Aljofan and Amirah M. Almokhtar. Formal analysis: Haya Alshutayli, Sara Alnowaiser and Albatool Falah Aljofan. Resources: Shaker O. Alreshidi and Taghreed Alnahedh. Writing – original draft: Haya Alshutayli, Sara Alnowaiser and Albatool Falah Aljofan. Writing – review and editing: All authors.

## Disclosure

The lead author Haya Alshutayli affirms that this manuscript is an honest, accurate, and transparent account of the study being reported; that no important aspects of the study have been omitted; and that any discrepancies from the study as planned (and, if relevant, registered) have been explained.

## Conflicts of Interest

The authors declare no conflicts of interest.

## Data Availability

The data that support the findings of this study are available from the corresponding author upon reasonable request, in accordance with ethical and privacy considerations.
